# Leucocyte subset-specific type 1 interferon signatures in SLE and other immune-mediated diseases

**DOI:** 10.1136/rmdopen-2015-000183

**Published:** 2016-05-16

**Authors:** Shaun M Flint, Vojislav Jovanovic, Boon Wee Teo, Anselm Mak, Julian Thumboo, Eoin F McKinney, James C Lee, Paul MacAry, David M Kemeny, David RW Jayne, Kok Yong Fong, Paul A Lyons, Kenneth GC Smith

**Affiliations:** 1 Department of Medicine, The University of Cambridge, Cambridge, UK; 2 Cambridge Institute of Medical Research, The University of Cambridge, Cambridge, UK; 3 Immunology Programme and Department of Microbiology Centre for Life Sciences, National University of Singapore, Singapore, Singapore; 4 Department of Medicine, Yong Loo Lin School of Medicine, National University of Singapore, Singapore, Singapore; 5 Department of Rheumatology and Immunology, Singapore General Hospital, Singapore, Singapore

**Keywords:** Systemic Lupus Erythematosus, Autoimmunity, Cytokines

## Abstract

**Objectives:**

Type 1 interferons (IFN-1) are implicated in the pathogenesis of systemic lupus erythematosus (SLE), but most studies have only reported the effect of IFN-1 on mixed cell populations. We aimed to define modules of IFN-1-associated genes in purified leucocyte populations and use these as a basis for a detailed comparative analysis.

**Methods:**

CD4+ and CD8+ T cells, monocytes and neutrophils were purified from patients with SLE, other immune-mediated diseases and healthy volunteers and gene expression then determined by microarray. Modules of IFN-1-associated genes were defined using weighted gene coexpression network analysis. The composition and expression of these modules was analysed.

**Results:**

1150 of 1288 IFN-1-associated genes were specific to myeloid subsets, compared with 11 genes unique to T cells. IFN-1 genes were more highly expressed in myeloid subsets compared with T cells. A subset of neutrophil samples from healthy volunteers (HV) and conditions not classically associated with IFN-1 signatures displayed increased IFN-1 gene expression, whereas upregulation of IFN-1-associated genes in T cells was restricted to SLE.

**Conclusions:**

Given the broad upregulation of IFN-1 genes in neutrophils including in some HV, investigators reporting IFN-1 signatures on the basis of whole blood samples should be cautious about interpreting this as evidence of *bona fide* IFN-1-mediated pathology. Instead, specific upregulation of IFN-1-associated genes in T cells may be a useful biomarker and a further mechanism by which elevated IFN-1 contributes to autoimmunity in SLE.

**Key messages**

**What is already known about this subject?**

- Upregulation of type 1 interferon (IFN-1)-associated genes in peripheral blood leucocytes of patients with systemic lupus erythematosus (SLE) is well described, but studies to date primarily use mixed cell populations.

**What does this study add?**

- This study finds differences in the pattern of upregulation of IFN-1-associated genes between the major leucocyte subsets (CD4+ and CD8+ T cells, monocytes and neutrophils).
- A subset of neutrophil samples displayed increased IFN-1-associated gene expression in healthy volunteers and conditions not classically associated with IFN-1, whereas upregulation of IFN-1-associated genes in T cells was found to be much more specific for SLE.

**How might this impact on clinical practice?**

- Whole blood assays of IFN-1-associated gene expression should be avoided because of the lack of specificity of the upregulation of IFN-1-associated genes and the potential for confounding by changes in whole blood cellular composition.

## Introduction

Systemic lupus erythematosus (SLE) has been linked to markedly increased levels of circulating type 1 interferons (IFN-1) since the late 1970s. IFN-1 are a group of related cytokines with potent capacity to initiate an antiviral response. Therapeutic interferon-α neutralising monoclonal antibodies are under active evaluation and much effort has gone into defining the coordinated effects of IFN-1 on whole blood and peripheral blood mononuclear cells (PBMC).^1^ Less is known about the effects of IFN-1 on gene expression in individual immune cell populations in SLE.

The primary action of IFN-1 on myeloid cells in vitro is to stimulate the activation and differentiation of dendritic cells, inducing them to upregulate class 1 major histocompatibility complex (MHC) and costimulatory molecules.^2^ Their action on T cells, however, is context dependent. For example, prolonged exposure to IFN-1 prior to activation inhibits proliferation, whereas T cells exposed to IFN-1 at activation mount a robust proliferative response. Although IFN-1 acts classically to promote an antiviral TH1 response, in other settings IFN-1 demonstrably inhibit both TH1 and TH17 responses.^3–5^ Which of these effects of IFN-1 are dominant in immune cell populations in SLE remains unclear. Certainly, a broad activation signature has been described in the CD4+ T cell in SLE, whereas IFN-1-inducible gene expression in monocytes more closely mirrors that observed for PBMC.^6^ However, this is based on small studies with subset-specific IFN-1-inducible genes either defined by their upregulation in SLE or by short-term IFN-1 stimulation experiments, potentially missing effects related to the leucocyte subset or chronicity of IFN-1 signalling.^6–8^

We use an unsupervised, data-driven approach to define modules of IFN-1-associated genes in individual leucocyte subsets. This distinguishes it from similar prior studies such as that of Lyons *et al*,^6^ which instead compare lists of genes differentially expressed in SLE with published gene lists to infer IFN-1 signalling. These modules then form the basis for an analysis of IFN-1-associated gene expression, in which we show that while IFN-1 genes are upregulated in neutrophils in a subset of patients across a range of immune-mediated diseases and some healthy volunteers, upregulation of IFN-1-associated genes in T cells is largely restricted to patients with SLE.

## Methods

### Study populations

Consenting patients with SLE (American College of Rheumatology (ACR) classification)^9^ receiving minimal immunosuppression (ie, <10 mg prednisolone/day or <50 mg azathioprine/day, without rituximab or cytotoxics within the preceding 3 months), anti-neutrophil cytoplasmic antibody (ANCA)-associated vasculitis (AAV) and inflammatory bowel disease (IBD) were recruited during active disease (as described in references 10 and 11). Patients with Behçet's syndrome were also recruited during active disease (see online supplementary tables S1 and S2). Patients with SLE were also recruited from the National University Hospital in Singapore to the same criteria, and a further cross-sectional cohort from a rheumatology outpatient clinic at Singapore General Hospital.^12^ Ethical approval for the Cambridge cohort was from East of England—Cambridge Central Research Ethics Committee and for the Singapore cohort was from the Domain Specific Review Board of the National Healthcare Group. Local, ethnically matched healthy volunteers were also recruited at each centre.

10.1136/rmdopen-2015-000183.supp1 Supplementary data

### Sample processing

Blood samples were processed as described.^12^ In brief, PBMC were obtained using density gradient centrifugation. Half underwent sequential positive selection using CD14+ and CD4+ microbeads (Miltenyi) to yield monocytes and CD4+ T cells. The remainder underwent positive selection using CD19+ and CD8+ microbeads (Miltenyi) to yield CD8+ T cells. Neutrophils were isolated from the red cell pellet by lysis followed by CD16+ microbead (Miltenyi) selection. Separation purities were monitored using flow cytometry as previously reported.^10^ Lysed samples were kept in RLT buffer (Qiagen) at −80°C until required, and then RNA was extracted using the AllPrep Mini kit (Qiagen) and hybridised to Affymetrix HuGene 1.1 microarrays according to the manufacturer's protocols. Singaporean samples were processed in Singapore using the same protocol and then shipped to Cambridge as lysed samples in RLT buffer, with comparable separation purities and RNA quality.

### Bioinformatics

Microarrays were preprocessed together and gene modules derived using weighted gene coexpression network analysis (WGCNA) (see online supplementary information).^13^ Multiple disease cohorts were included in order to improve the specificity of the resulting IFN-1 gene modules in each leucocyte subset, which were identified by comparison to the signature in Yao *et al*,^14^ chosen for its specificity as described in the online supplementary information. An ‘interferon score’ for each module was defined as the first principal component of scaled expression of module genes. The expression of genes belonging to IFN-1 modules in all cell subsets was explored using hierarchical clustering (scaled by gene) with a Euclidean distance metric.

### Other statistical methods

All data analysis was performed in R (http://www.r-project.org). As an exploratory analysis, no formal sample size estimation was required. Non-parametric statistics were used, with an α value of 0.05 being considered significant.

## Results

### Leucocyte subset-specific IFN-1 gene modules

In total, 1065 gene expression arrays from 385 discrete patients across seven immune-mediated conditions and four leucocyte subsets were included in the analysis (see online supplementary tables S1 and S2). WGCNA was applied to this data set to identify gene modules of coexpressed genes within each leucocyte subset as described in the online supplementary information. In brief, genes were assigned to modules using hierarchical clustering applied to a distance metric based on weighted gene–gene correlations. A measure of the degree to which a given gene belongs to a given module (ie, module membership score) was obtained by considering the correlation of a gene's individual expression profile with that of the module overall. Even though genes are assigned to modules based on similarity of expression profile, within each module there is still usually a range of module membership scores.

**Figure 1:**
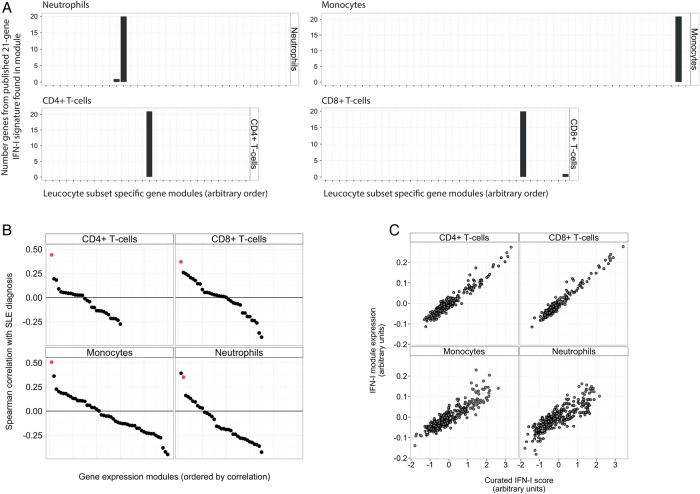
The identification of leucocyte subset-specific type 1 interferon (IFN-1) modules. (A) A bar chart showing the number of genes belonging to a published 21-gene IFN-1 signature^14^ contained in each of the gene modules identified by the weighted gene coexpression network analysis (WGCNA) algorithm in each leucocyte subset. (B) Spearman correlation coefficient for the correlation of gene modules in each leucocyte subset with a diagnosis of systemic lupus erythematosus (SLE) (coded 0,1). The IFN-1-associated gene module is highlighted in red. (C) For all samples in the analysis, regardless of diagnosis, the correlation of IFN-1-associated module expression with a curated interferon score based on the mean expression of genes in the published 21-gene IFN-1 signature.

Within each leucocyte subset, one module clearly represented IFN-1 gene expression (ie, IFN-1 module). This was identified by (1) the number of genes from a published 21-gene IFN-1 signature, chosen for its specificity, that were included in the module and (2) the association of increased module expression with a diagnosis of SLE (figure 1A, B). As an additional check, we confirmed that IFN-1 module expression correlated strongly with a curated IFN-1 score (ie, the mean expression of genes in the 21-gene IFN-1 signature, using data from the same leucocyte subset, figure 1C). Although a second module of 26 genes in neutrophils also correlated well with a diagnosis of SLE (figure 1B), it did not correlate well with the curated IFN-1 score (Spearman r=0.23) and only contained genes suggestive of broader cellular activation (ie, *FOS, JUN, CCL3, CCL4, CXCL1, CXCL8, PTGS2*).

**Figure 2:**
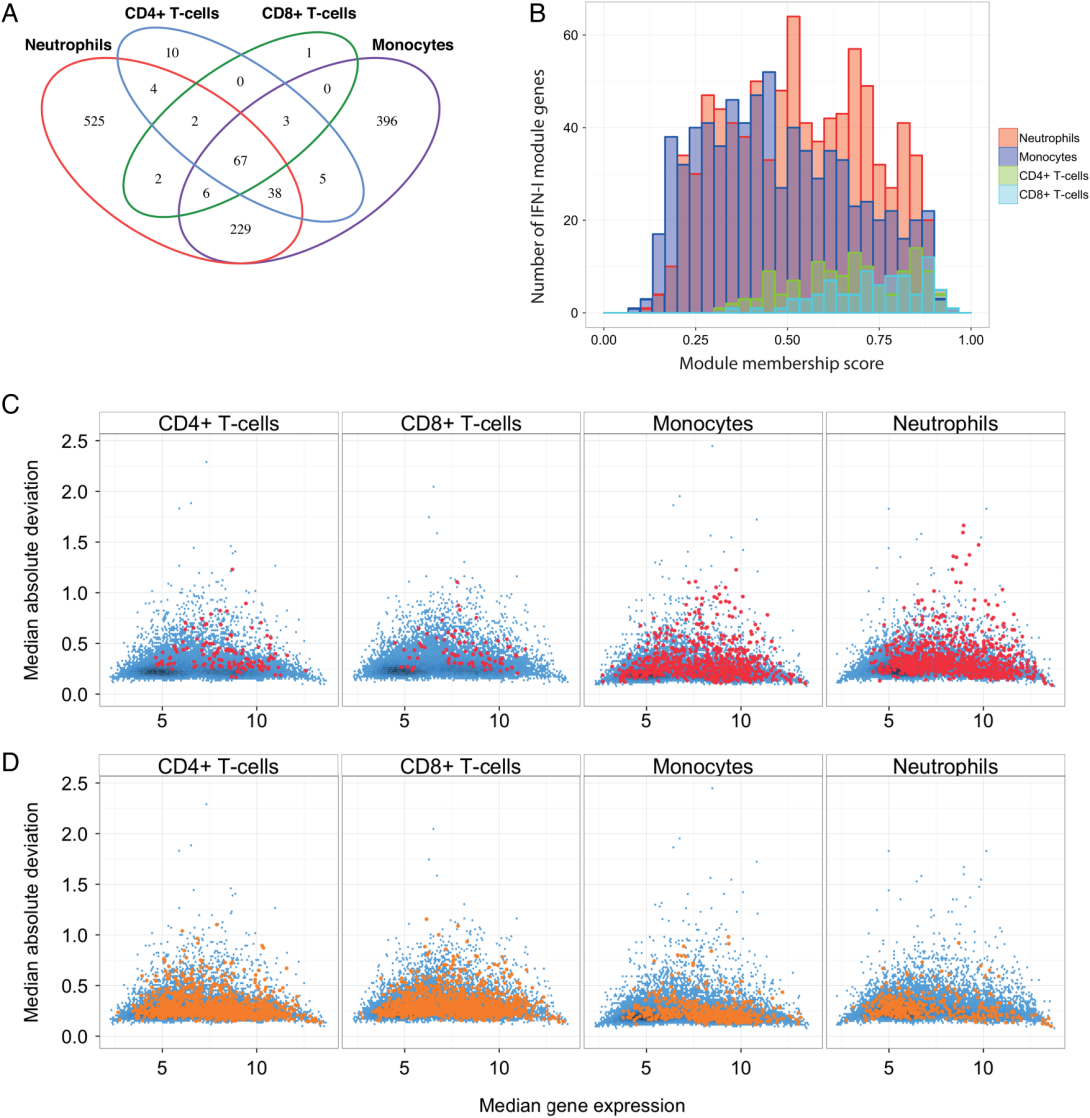
Properties of leucocyte subset-specific type 1 interferon (IFN-1) modules. (A) A Venn diagram showing overlap in membership of each of the four leucocyte subset-specific IFN-1 gene modules. (B) Distribution of module membership scores for genes in each of the leucocyte subset-specific IFN-1 modules. (C) MA plots depicting median absolute deviation (MAD) against median gene expression for genes in each of the subset-specific IFN-1 modules (red points). The distribution of MAD against median gene expression for all genes in the analysis is shown in blue. (D) MA plots as in (C), except that they depict the MAD versus median expression for genes belonging to at least one IFN-1 module, but not in the leucocyte subset shown (yellow points). Expression values are expressed in arbitrary units.

Overall, 1288 genes belonged to an IFN-1 module in at least one leucocyte subset, with module assignments and module membership scores listed in online supplementary table S3. Sixty-seven of these 1288 genes were IFN-1-associated in all (‘core IFN-1 genes’), whereas the majority of IFN-1-associated genes were unique to myeloid subsets (1150 genes), compared with only 11 genes unique to T cells (*ABCG2*, *CYP2J2*, *FCRL3*, *IKBKE*, *IRS1*, *LINC01260*, *PSD3*, *RBMS3*, *TRAK2*, *YEATS2*, *NKAIN1*; figure 2A). Although this difference was partly driven by substantially more genes with lower module membership scores in the myeloid IFN-1 modules (ie, lower correlation with IFN-1 expression), for any given level of module membership score there were more genes in the myeloid IFN-1 modules than in the T cell IFN-1 modules (figure 2B). In other words, a broader range of genes were upregulated in vivo by chronic IFN-1 exposure in myeloid leucocyte subsets than in T cells.

We considered the possibility that the substantially smaller T cell IFN-1 modules reflected a lack of expression of IFN-1 genes in this cell subset. Although gene microarrays return a relative, not absolute, measure of expression, non-expressed genes tend to have low variance across samples and lower expression levels. Variance (as a mean absolute deviation) and median expression are plotted in figure 2C for genes belonging to the IFN-1 module in each leucocyte subset (red) against a background of all genes (blue). Figure 2D shows the same for genes belonging to an IFN-1 module in at least one leucocyte subset but not the leucocyte subset depicted. As can be seen, many IFN-1 genes that are not IFN-1 associated in CD4+ and CD8+ T cells (ie, figure 2D, first 2 panels) would still appear to have T cell expression.

Subset-specific IFN-1 gene modules were also compared with published whole blood IFN-1 gene modules, particularly those of Chiche *et al*.^15^ We found that the differing thresholds for expression of the whole blood modules described in that study might also relate to differences in the leucocyte subset-specific transcriptional response to IFN-1 as well as differences in response to IFN-1 and IFN-2, as hypothesised (see online supplementary figure S1).

### Core IFN-1 genes are more highly expressed in myeloid subsets

**Figure 3:**
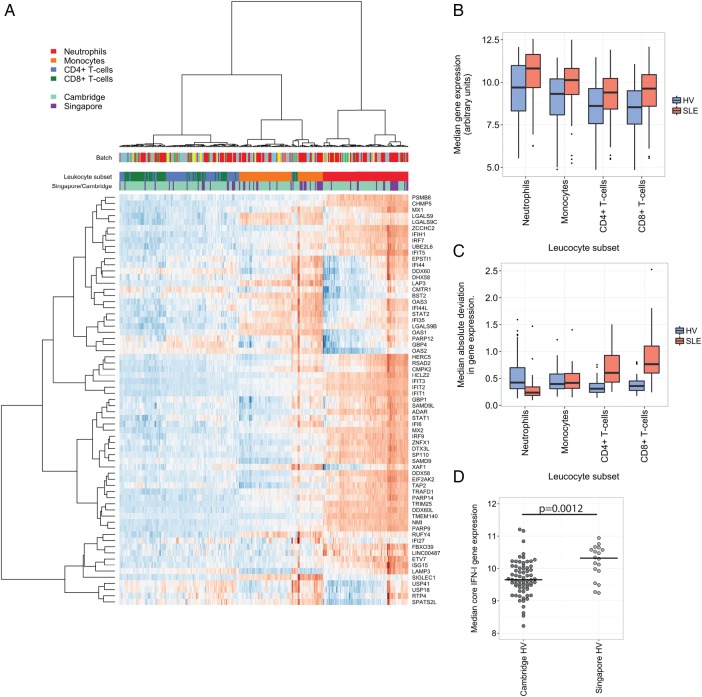
Core type 1 interferon (IFN-1)-associated genes are more highly expressed in myeloid subsets. (A) A heat map showing the scaled (by gene) expression in healthy volunteers of 67 core IFN-1 genes (ie, genes belonging to IFN-1 modules in all leucocyte subsets). Samples are in columns and genes in rows. Order in each is determined by hierarchical clustering (Ward's method) using a Euclidean distance metric. Leucocyte subset, cohort and array batch are shown as coloured bands above the main heat map. Box plots showing the distribution of median gene expression values (B) and median absolute deviation (C) for each of 67 core IFN-1 genes in healthy volunteers and patients with systemic lupus erythematosus (SLE), stratified by leucocyte subset. (D) Median core IFN-1 gene expression in neutrophil samples, stratified by centre. Wilcoxon p value is shown.

We sought to identify patterns in the expression of the 67 core genes belonging to IFN-1 modules in each leucocyte subset. First, we examined their basal expression in healthy volunteers. Using only the scaled expression of these 67 genes, we found that lymphocyte, monocyte and neutrophil samples clustered separately, reflecting the higher expression of many core IFN-1 genes in myeloid, and particularly neutrophil, subsets (figure 3A). We confirmed this by examining the median expression of these genes by leucocyte subset and diagnosis (figure 3B). Median expression of core IFN-1 genes was increased in myeloid subsets compared with lymphoid subsets in healthy volunteers (HV) and SLE, with median expression in SLE being greater than that in HV for each leucocyte subset. We also examined the range of expression of these core IFN-1 genes, finding that CD4+ and CD8+ T cells from SLE samples had a much greater range of expression than when compared with HV and with SLE myeloid cells (figure 3C). These differences remained when stratified by centre (see online supplementary figure S2). In a subanalysis, we found that the median core IFN-1 gene expression was greater in neutrophils from healthy volunteers in Singapore than in Cambridge (figure 3D, see online supplementary figure S3), but as samples were not prospectively matched by centre, this observation requires validation (see online supplementary table S4).

10.1136/rmdopen-2015-000183.supp2 Supplementary table 3

### Specificity of the IFN-1 signature varies by leucocyte subset

**Figure 4:**
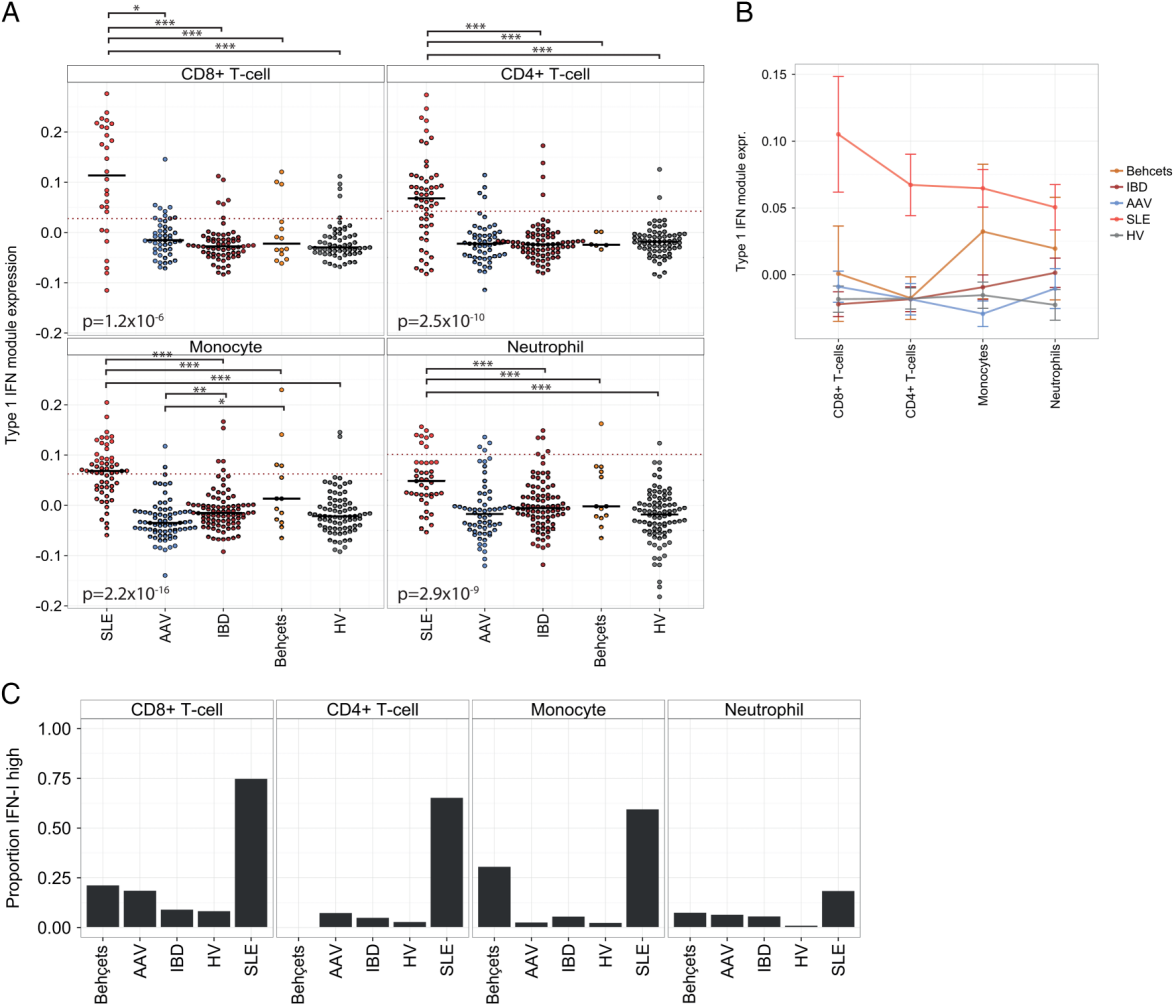
The specificity of an IFN-1 signature varies by leucocyte subset. (A) IFN-1 module expression by diagnosis for the four leucocyte subsets. Short black horizontal lines indicate the median of each group, and the dotted red horizontal lines indicate the threshold used for elevated IFN-1 module expression (see text). p Values (Kruskal-Wallis) are shown in each panel, testing for differences by diagnosis. Where differences are significant overall, significant pairwise differences (Wilcoxon test, with Holm correction) are shown above. *p<0.05, **p<0.005, ***p<0.0005. (B) Mean and SEM is shown for the same data as (A), stratified by diagnosis and leucocyte subset. (C) Proportion of samples within each diagnostic category with elevated IFN-1 module expression. IBD, inflammatory bowel disease; IFN-1, type 1 interferon; SLE, systemic lupus erythematosus.

The IFN-1 module expression in each leucocyte subset was compared between immune-mediated conditions. Increased module expression was observed in most, but not all, of the SLE samples regardless of leucocyte subset. However, the upregulation of IFN-1 module expression in CD4+ and CD8+ T cell samples from patients with SLE compared with other samples was proportionally greater than that observed in myeloid subsets, resulting in a cleaner separation of these SLE samples from other diagnoses (figure 4A, B). This partly reflects a greater spread of IFN-1 module expression in monocyte and neutrophil samples across all diagnostic categories, to the extent that a subset of neutrophil and monocyte samples from healthy volunteers and patients with IBD, AAV and Behçet's syndrome displayed IFN-1 module expression levels comparable to that of SLE samples (figure 4A, B). We explored whether this was confounded by differing IFN-1 inducible genes in each subset, or by differing samples used in each cell subset, and found neither of these factors to be important (see online supplementary figure S4A, B).

A threshold for elevated IFN-1 module expression in each leucocyte subset was set on the basis of the 99th centile of IFN-1 module expression in HV (after outliers, defined as those samples with an adjusted normal p value <0.001, were removed), as shown by the dashed lines in figure 4A. We found that by this method the proportion of SLE samples considered IFN-1 high varied considerably from 75% for CD8+ T cell samples to 19% for neutrophils. In each subset, a variable, but small, proportion of samples from patients with other diagnoses was also found to be IFN-1 high.

## Discussion

In this in vivo study of IFN-1-associated genes in T cells, monocytes and neutrophils, we found higher expression of core IFN-1-associated genes in neutrophils and monocytes, compared with T cells in healthy volunteers and in SLE. We also found that neutrophils and monocytes specifically upregulate a substantially broader range of genes than T cells in response to circulating IFN-1. Conversely, we found that IFN-1 module expression in patients with SLE overlapped that of patients with Behçet's syndrome, AAV and IBD in neutrophil and monocyte samples to a much greater extent than in T cells. The overlap appeared due to a broader range of IFN-1 module expression in neutrophil samples from healthy volunteers and diseases not usually thought to be IFN-1 mediated (ie, AAV^6^ ^16^). This meant that comparatively few neutrophil samples were considered IFN-high when using a cut-off based on the upper limit of IFN-1 module expression in healthy volunteers. Given the high sensitivity of this subset to a range of inflammatory stimuli, we wonder whether this may reflect the ability of even small levels of circulating IFN-1 to stimulate the downstream transcription of neutrophil IFN-1-associated genes.^17^ ^18^

Indeed, our finding of increased core IFN-1-associated gene expression in healthy volunteer myeloid samples compared with T cells is consistent with studies highlighting the importance of basal IFN-1 signalling for maintaining myeloid populations and for priming an effective innate immune response; studies of *Ifnar* and *Ifnb* knockout mice would suggest that basal IFN-1 signalling appears less important for T cell populations.^19^ ^20^ We hypothesise that prior priming by basal IFN-1 signalling is one factor that allows myeloid cells to rapidly mobilise a broader range of IFN-1 genes and generate higher gene expression levels of core IFN-1-associated genes compared with T cells.

In our cohort, we found that IFN-1 gene expression in neutrophils was higher in Singaporean healthy volunteers than their UK-based counterparts. While as a post hoc subanalysis it requires replication, this is an interesting observation, given that South-East Asia is a region with an increased prevalence of SLE: increased IFN-1 signalling in healthy volunteers may predispose to the development of SLE, as has been recently described for type 1 diabetes mellitus.^21^

The highest levels of IFN-1 module expression in T cells were only found in SLE. Given the well-described association between hypomethylation and increased gene expression, this would be consistent with studies reporting DNA hypomethylation near IFN-1-associated genes in CD4+ T cells from patients with SLE.^22^ Conceivably, increased IFN-1 module expression in T cells may also reduce the threshold for their activation, facilitating loss of tolerance and providing another mechanism by which elevated IFN-1 contributes to the development of autoimmunity in SLE. Regardless of the mechanism, this observation suggests that a T cell-specific assay of IFN-1 gene expression may provide a cleaner read-out of IFN-1 exposure in SLE than a whole blood assay.

This analysis extends a small number of prior studies examining IFN-1 in leucocyte subpopulations by considering additional leucocyte subsets and using more samples.^7^ ^23^ By using WGCNA to define IFN-1 modules, we avoided assumptions about module size or composition and additional disease cohorts allowed us to study IFN-1 gene expression across a range of conditions. Finally, we used a protocol shown to minimally affect in vivo gene expression; some prior studies of IFN-1 gene expression in leucocyte subsets in SLE used a protocol that, by maintaining cells in culture overnight, may have introduced substantial ex vivo differences and flattened existing ones.^23^

In summary, we found that although the neutrophil transcriptional response to IFN-1 involved the largest number of genes and the highest expression of core IFN-1-associated genes, IFN-1 gene expression in neutrophils lacked specificity for traditionally IFN-1-mediated conditions. We also found that an interferon signature in T cells was comparatively restricted to SLE samples, hypothesising that this may contribute to the loss of tolerance in this condition. The clinical significance of these observations is that researchers and clinicians reporting IFN-1 signatures on the basis of whole blood samples (with a large and variable proportion of neutrophils present) should be cautious about interpreting this as evidence of *bona fide* IFN-1-mediated pathology.^24^ ^25^ Instead, we would argue that the presence of an IFN-1 signature should be determined using at least PBMC, or perhaps even separated T cells.
