# Supplementary material for: Leucocyte subset-specific type 1 interferon signatures in SLE and other immune-mediated diseases
Source: RMD Open. 2016 May 16;2(1):e000183. doi: 10.1136/rmdopen-2015-000183 (PMC4879345; doi:10.1136/rmdopen-2015-000183)
Supplement: Supplementary data [file rmdopen-2015-000183supp.pdf]

Leukocyte subset-specific type 1 interferon signatures in SLE and other immune-mediated diseases.

Shaun M Flint, Vojislav Jovanovic, Boon Wee Teo, Anselm Mak, Julien Thumboo,
Eoin F McKinney, James C Lee, Paul MacAry, David M Kemeny, David RW Jayne, Kok Yong Fong, Paul A Lyons, Kenneth GC Smith*

Supplementary information

Microarray preprocessing.

All aspects were performed within R (www.r-project.org) and Bioconductor (www.bioconductor.org), and all arrays were processed together as follows:

1. Individual .CEL files were read into an ExpressionFeatureSet R object using the oligo package.[1]
2. Individual microarray quality was assessed using the arrayQualityMetrics package and any array not passing QC was discarded.[2]
3. The ExpressionFeatureSet was then normalised and summarized using the oligo package's implementation of the Robust Multichip Average (RMA) pre-processing methodology. The arrays were summarised at the gene level (i.e. target='core').
4. Batch effects were corrected for using an in-house implementation of the ComBat algorithm.[3] Diagnosis and leukocyte subset were included as a covariates to avoid flattening any biologically relevant variation. Care was taken to avoid unbalanced batches and small batches, as experience has shown that this can skew ComBat output.
5. As the HuGene 1.1 arrays often contain more than one probeset per gene, in order to minimize multiple testing and to simplify the analysis the Expressionset object was reduced further to one probe per annotated gene, keeping the probe with the largest variance.

Weighted Gene Coexpression Network Analysis.

WGCNA is an algorithm optimised for identifying modules of co-expressed genes within a gene expression dataset; we used the implementation contained within the WGCNA module within R.[4] Having prepared a common gene expression dataset containing all arrays used in the study (in order to minimize technical variation between cell types), we then divided this into four separate datasets, each one containing expression data from a single leukocyte subset. WGCNA was run on each expression data from each leukocyte subset separately. The number of arrays from each diagnosis in each leukocyte subset are summarised in **Table S1**.

The most important user-determined parameter in WGCNA is the soft thresholding power. This is used in the process of generating a weighted adjacency matrix, and the lowest value that results in an approximately scale-free network topology is usually chosen. We implemented the approach recommended by the package authors using the pickSoftThreshold function of the WGCNA package, selecting soft thresholding powers as follows CD8=10, CD4=11, CD14=14, CD16=11.

Using these powers, a topological overlap matrix (TOM) was generated from gene expression data using the TOMsimilarityFromExpr function of the WGCNA package (network type = "signed"). The TOM is a matrix of gene-gene similarities that is amenable to clustering. To facilitate clustering, we transformed the TOM into a dissimilarity matrix using the transformation 1-TOM.

Finally, modules of coexpressed genes were defined by average linkage hierarchical clustering. We used the dynamic tree cut routine as implemented within the WGCNA package in R to choose the boundaries be-

tween individual modules (parameters: method='hybrid', minimum module size=25, deepSplit=2, PAM respects dendrogram=T).[5] This routine defines modules based on the shape of the dendrogram, and is more flexible than choosing a single cutoff value.

The outcome of this procedure was a separate series of modules (i.e. 'clusters' or 'groups') of coexpressed genes for each leukocyte subset. The module representing the transcriptional response to type 1 interferon (IFN-I) was selected by comparison to the 21 gene IFN-I 'signature' (*IFI27, IFI6, RSAD2, IFI44, IFI44L, USP18, LY6E, OAS1, SIGLEC1, ISG15, IFIT1, OAS3, HERC5, MX1, LAMP3, EPSTI1, IFIT3, OAS2, RTP4, PLSCR1, SPATS2L*) published in [6]. This signature was chosen for its specificity: it consists of genes upregulated *in vitro* after healthy PBMC were cultured with a range of IFN-I subtypes (10 IFN-α subtypes and IFN-β) that were also upregulated in the whole blood of SLE patients and that were neutralised by an anti-IFN-α monoclonal antibody.

Relationship of leukocyte subset-specific IFN-I modules to published IFN-I analyses.

A recent publication has reported three distinct clusters of IFN-I associated gene expression in whole blood, annotated as modules 1.2, 3.4 and 5.12.[7] Module 1.2 was noted to be most sensitive to IFN-I expression, being upregulated earlier and to a higher degree than the other modules, whereas genes in module 5.12 were only upregulated in the subset of patients possessing the strongest IFN-I signature. The authors presented some evidence that this may represent differing sensitivities of genes in each module to circulating interferon-β and interferon-γ. We wondered whether these patterns might also be explained by their expression within lymphoid and myeloid subsets of varying size.

First, we examined the overlap between genes in the published whole blood modules 1.2, 3.4 and 5.12 and the 67 core IFN-I genes belonging to IFN-I modules in all leukocyte subsets. Fifteen (68%) of genes in the whole blood module 1.2 were also members of this core IFN-I gene set, compared with 18 (42%) of genes in module 3.4 and 13 (28%) of genes in module 5.12. The differing proportions were statistically significant ($\chi 2 = 9.8$, p-value = 0.0075). We then examined the overlap between genes in the published whole blood modules 1.2, 3.4 and 5.12 and each individual leukocyte subset-specific IFN-I module. The module membership scores for overlapping genes are shown in figure S3. For CD4+ and CD8+ T-cells in particular, we found that module membership scores for genes shared with module 1.2 were higher than those shared with modules 3.4 and 5.12. This suggests that genes in module 5.12 are more weakly associated with IFN-I in T-cells than those in module 1.2 and that this, combined with differing poportions of T-cells and myeloid cells, may represent an alternative explanation the different properties of the whole blood IFN-I associated modules 1.2, 3.4 and 5.12.

1. Carvalho B, Bengtsson H, Speed TP, Irizarry RA. Exploration, normalization, and genotype calls of high-density oligonucleotide SNP array data. Biostatistics. 2007;8:485–499.
2. Kauffmann A, Gentleman R, Huber W. arrayQualityMetrics - A bioconductor package for quality assessment of microarray data. Bioinformatics. 2009;25(3):415–416.
3. Johnson WE, Li C, Rabinovic A. Adjusting batch effects in microarray expression data using empirical Bayes methods. Biostatistics. 2007;8(1):118–127.
4. Langfelder P, Horvath S. WGCNA: an R package for weighted correlation network analysis. BMC Bioinformatics. 2008;9:559.
5. Langfelder P, Zhang B, Horvath S. Defining clusters from a hierarchical cluster tree: The Dynamic Tree Cut package for R. Bioinformatics. 2008;24(5):719–720.
6. Yao Y, Higgs BW, Morehouse C, et al. Development of Potential Pharmacodynamic and Diagnostic Markers for Anti-IFN-α Monoclonal Antibody Trials in Systemic Lupus Erythematosus. Hum Genomics Proteomics 2009;2009. doi:10.4061/2009/374312
7. Chiche L, Jourde-Chiche N, Whalen E, et al. Modular transcriptional repertoire analyses of adults with systemic lupus erythematosus reveal distinct type I and type II interferon signatures. Arthritis Rheumatol. 2014;66(6):1583–95.

Supplementary Tables

Table S1. Number of gene expression microarrays by diagnosis and leukocyte subset.

Table S2. Overall patient demographics.

Table S3. IFN-I module genes, with membership and module membership scores for each leukocyte subset. (supplied as separate .xls spreadsheet)

Table S4. Healthy volunteer demographics by center (neutrophil samples).

Table S1. Number of gene expression microarrays by diagnosis and leukocyte subset.

	SLE	AAV	Behçet's	IBD	Healthy	Total
CD4 + T-cells n (%)	55 (21%)	53 (21%)	6 (2.3%)	78 (30%)	66 (26%)	258
CD8 + T-cells n (%)	28 (13%)	48 (22%)	14 (6.5%)	65 (30%)	59 (28%)	214
Monocytes n (%)	57 (19%)	72 (24%)	13 (4.2%)	87 (28%)	77 (25%)	306
Neutrophils n (%)	43 (15%)	60 (21%)	13 (4.5%)	86 (30%)	85 (30%)	287

Table S2. Overall patient demographics

	CD4+ T-cells			CD8+ T-cells			Monocytes			Neutrophils		
	SLE	non-SLE	Healthy	SLE	non-SLE	Healthy	SLE	non-SLE	Healthy	SLE	non-SLE	Healthy
N	55	137	56	28	127	59	57	172	77	43	159	85
Median age (IQR) years	40 (33-51)	46 (29-61)	43 (30-54)	42 (35-50)	46 (31-61)	47 (31-54)	39 (31-48)	46 (30-62)	43 (27-53)	38 (32-46)	45 (29-61)	41 (27-52)
Female (%)	50 (91)	82 (60)	39 (59)	25 (89)	74 (58)	32 (54)	53 (93)	100 (58)	48 (62)	40 (93)	93 (58)	51 (60)
Home centre												
Cambridge	17	137	54	16	127	53	12	172	53	1	153	67
Singapore	38	-	12	12	-	6	45	-	24	42	-	18
ANA+ (%)	42 (91) missing = 9			22 (88) missing = 3			44 (92) missing = 9			33 (97) missing = 9		
dsDNA+ (%)	42 (86) missing = 6			16 (80) missing = 8			46 (90) missing = 6			41 (98) missing = 1		
ENA specificity	missing=28			missing = 15			missing = 22			missing = 14		
anti-Ro (%)	12 (44)			3 (23)			17 (49)			15 (52)		
anti-La (%)	6 (22)			0			8 (23)			8 (28)		
anti-RNP (%)	8 (30)			3 (23)			13 (37)			10 (34)		
anti-Sm (%)	4 (15)			3 (23)			8 (23)			9 (31)		
Cambridge Cohort												
median BILAG (IQR)	14 (17-21)			18 (12-21)			16 (12-20)			-		
Renal flare (%)	5 (33)			5 (33)			6 (55)			-		

Table S4. Healthy volunteer demographics by center (neutrophil samples).

	**Singapore**	**Cambridge**
**N**	**18**	**67**
**Median age (IQR), years**	**27 (24-41)**	**44 (31-55)***
**Female, (%)**	**16 (89%)**	**35 (52%)****

* $p = 0.0016$ (Wilcoxon ranksum test); ** $p = 0.01$ (Chi-squared test)

Supplementary Figures

Figure S1. Leukocyte subset-specific IFN-I module membership scores for genes in each of the three whole blood IFN-I modules described by Chiche et al [7] (annotated as modules 1.2, 3.4 and 5.12) that also overlap with leukocyte subset-specific IFN-I modules in this analysis. P-values (Kruskal-Wallis) are shown in each panel, testing for differences by module. Where differences are significant overall, significant pairwise differences (Wilcoxon test, with Holm correction) are shown above. * $p<0.05$, ** $p<0.005$, *** $p<0.0005$.

Figure S2. Box plots showing the distribution of median gene expression values for each of 67 core IFN-I genes in healthy volunteers and SLE patients, stratified by leukocyte subset and center.

Figure S3. Median expression of core type 1 inteferon associated genes in CD4+ and CD8+ T-cells and monocytes from HV, stratified by leukocyte subset. P-value is from a Wilcoxon rank-sum test.

Figure S4. (A) Type 1 IFN module expression by diagnosis for the four leukocyte subsets, represented as a parallel coordinates plot with lines joining samples from the same patient and bleed. **(B)** *OAS3* gene expression (log2 scale) shown by diagnosis for the four leukocyte subsets. Horizontal lines indicate the median of each group.

Figure S1

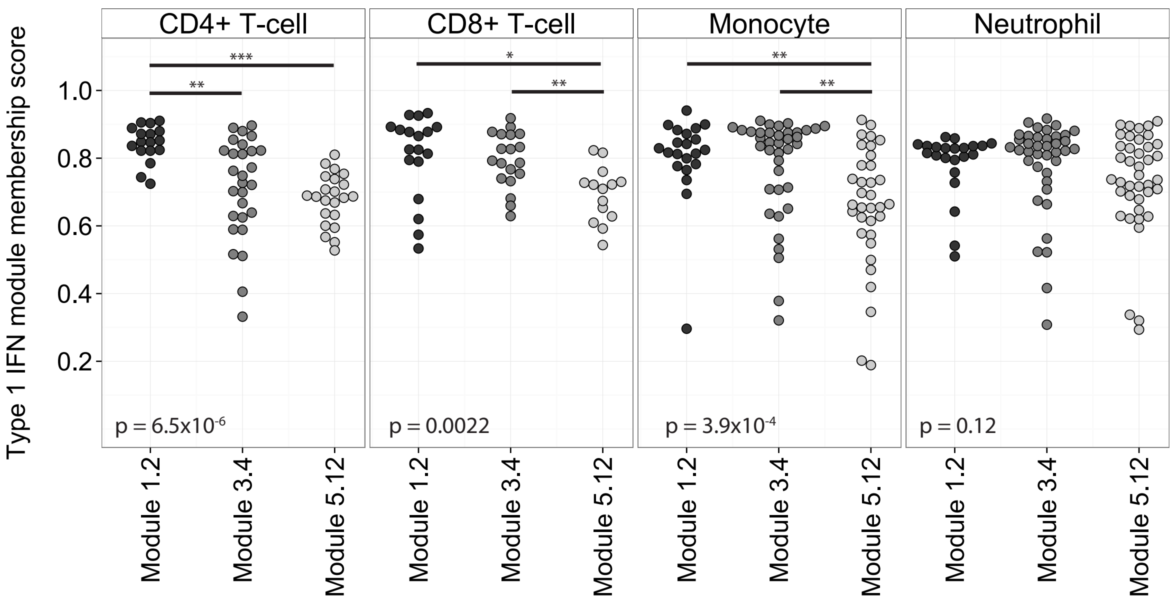

CD4+ T-cell
CD8+ T-cell
Monocyte
Neutrophil
Type 1 IFN module membership score
1.0
0.8
0.6
0.4
0.2
***
**
*
p = 6.5x10-6
p = 0.0022
p = 3.9x10-4
p = 0.12
Module 1.2
Module 3.4
Module 5.12

Figure S2

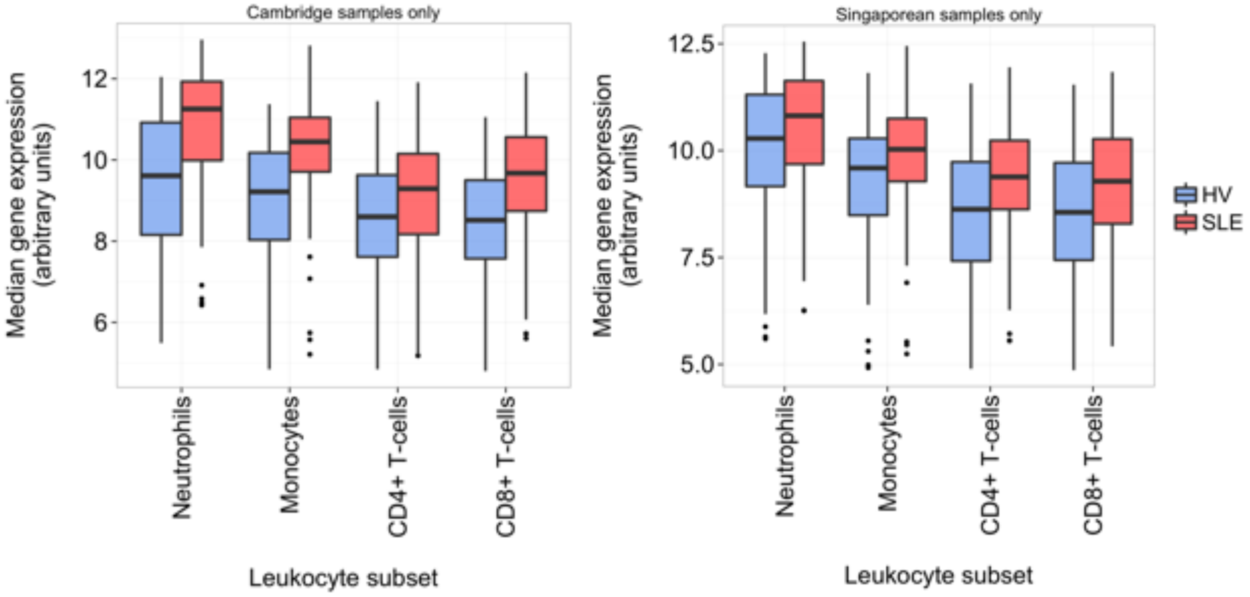

Figure S3

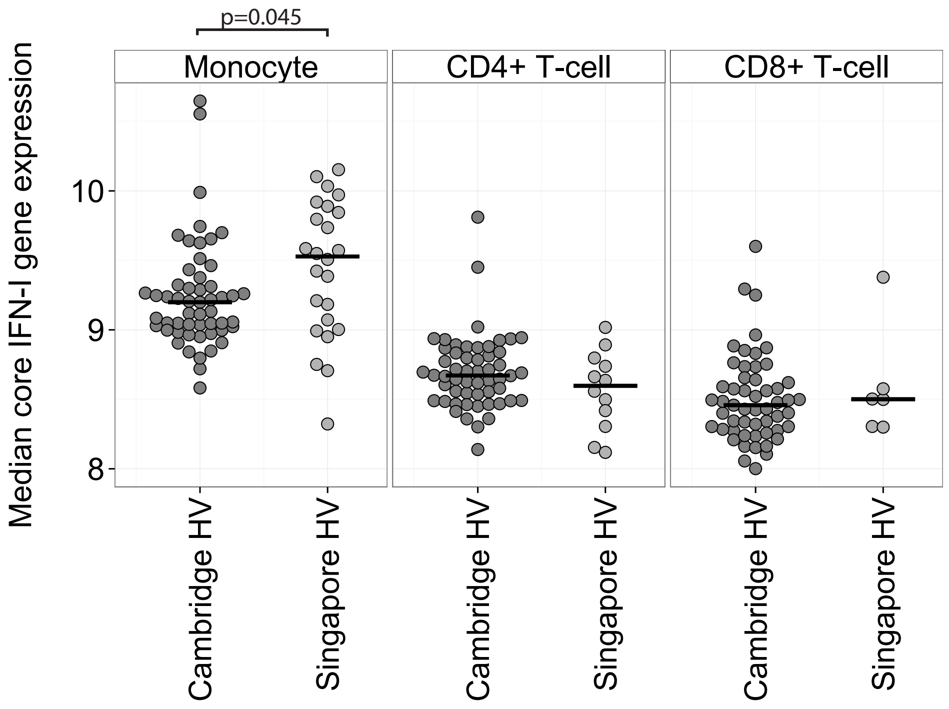

Figure S4

A

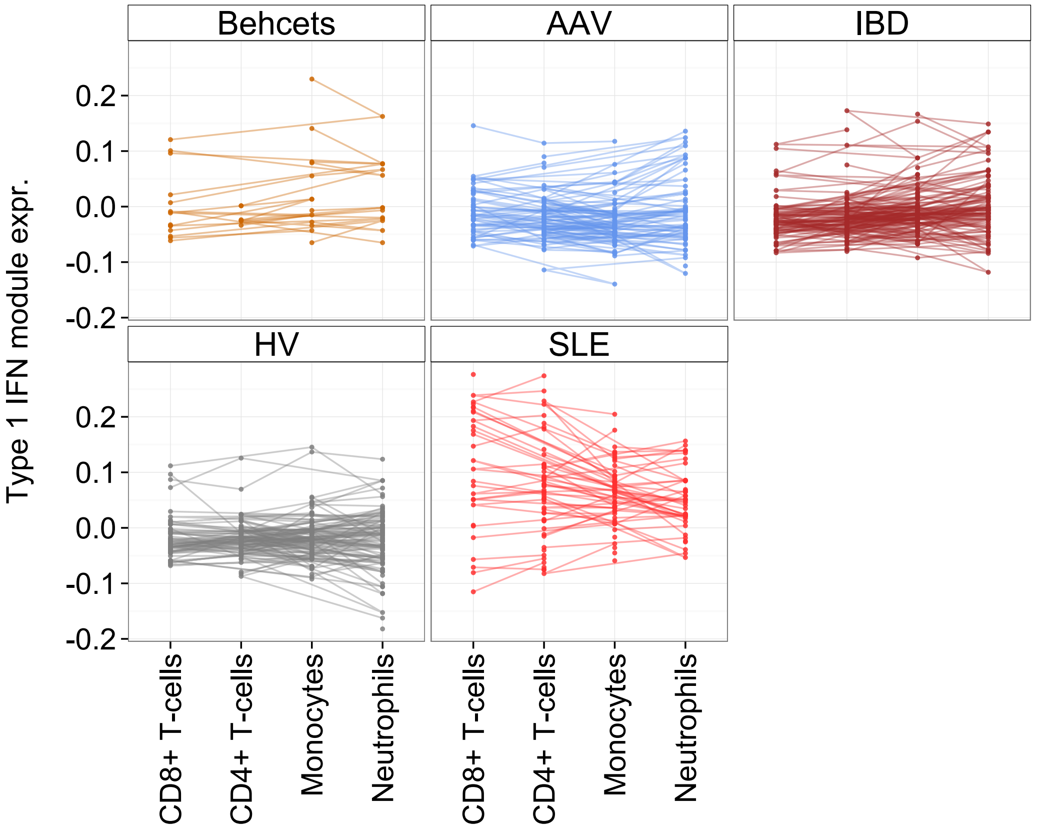

Behcets
AAV
IBD
HV
SLE
Type 1 IFN module expr.
0.2
0.1
0.0
-0.1
-0.2
CD8+ T-cells
CD4+ T-cells
Monocytes
Neutrophils

B

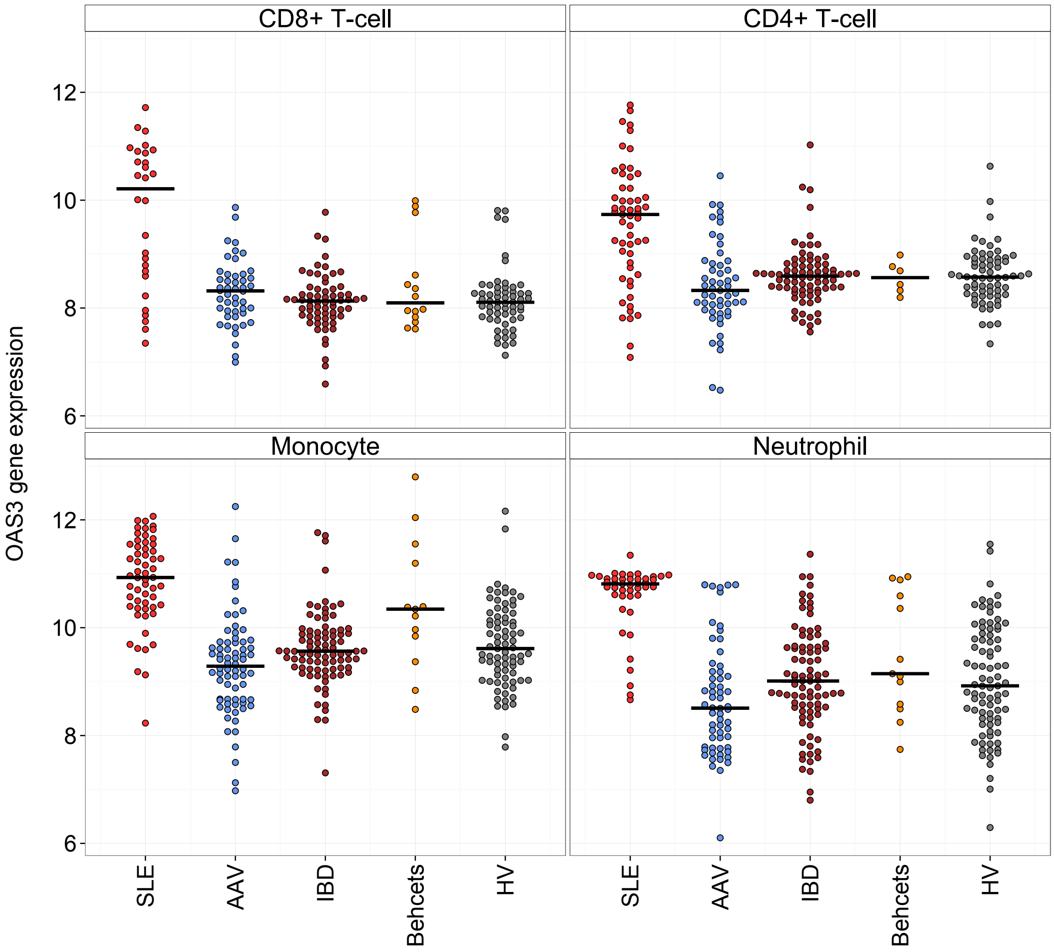

CD8+ T-cell
CD4+ T-cell
Monocyte
Neutrophil
OAS3 gene expression
12
10
8
6
SLE
AAV
IBD
Behcets
HV
